# A trait-based typification of urban forests as nature-based solutions

**DOI:** 10.1016/j.ufug.2022.127780

**Published:** 2022-12

**Authors:** Sebastian Scheuer, Jessica Jache, Martina Kičić, Thilo Wellmann, Manuel Wolff, Dagmar Haase

**Affiliations:** a Geography Department, Landscape Ecology Lab, Humboldt-Universität zu Berlin, Unter den Linden 6, 10099 Berlin, Germany; b Croatian Forest Research Institute, Division for International Scientific Cooperation in Southeast Europe, Cvjetno naselje 41, 10450 Jastrebarsko, Croatia; c UFZ – Helmholtz Centre for Environmental Research, Department of Computational Landscape Ecology, Permoserstr. 15, 04318 Leipzig, Germany; d UFZ – Helmholtz Centre for Environmental Research, Department of Urban and Environmental Sociology, Permoserstr. 15, 04318 Leipzig, Germany

**Keywords:** Urban forest, Nature-based solution, Typology, Trait-based modelling, Semantics, Ontology

## Abstract

Urban forests as nature-based solutions (UF-NBS) are important tools for climate change adaptation and sustainable development. However, achieving both effective and sustainable UF-NBS solutions requires diverse knowledge. This includes knowledge on UF-NBS implementation, on the assessment of their environmental impacts in diverse spatial contexts, and on their management for the long-term safeguarding of delivered benefits. A successful integration of such bodies of knowledge demands a systematic understanding of UF-NBS. To achieve such an understanding, this paper presents a conceptual UF-NBS model obtained through a semantic, trait-based modelling approach. This conceptual model is subsequently implemented as an extendible, re-usable and interoperable ontology. In so doing, a formal, trait-based vocabulary on UF-NBS is created, that allows expressing spatial, morphological, physical, functional, and institutional UF-NBS properties for their typification and a subsequent integration of further knowledge and data. Thereby, ways forward are opened for a more systematic UF-NBS impact assessment, management, and decision-making.

## Highlights

• A typology on urban forests as nature-based solutions is developed.
• Trait-based modelling to discern characteristics of urban forest types.
• Semantic modelling provides a highly systematic typology of urban forest.
• The typology is formalized as a machine-interpretable ontology.
• Formal knowledge enables re-use and the integration of knowledge and data.

## Graphical Abstract

**Figure:**
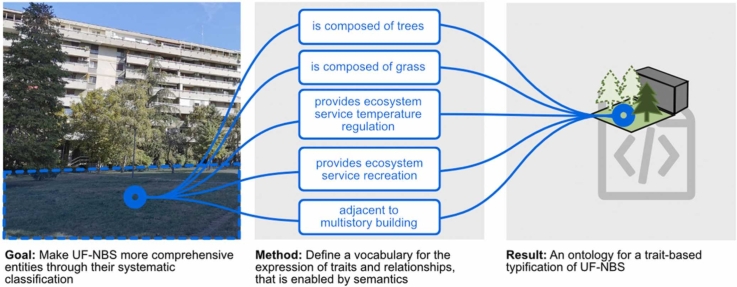

## 1 Introduction

Urban forest broadly refers to natural and planted trees in cities (Ordóñez and Duinker, 2010). It is understood as tree-dominated urban ecosystems (Berglihn and Gómez-Baggethun, 2021) comprising the entirety of woodlands, groups of trees, and individual trees on public and private land, including street trees, trees within parks or gardens, and even trees on facades or rooftops (Endreny, 2018, FAO, 2016, Konijnendijk, 2003, Ordóñez, 2021; cf. Table 1). Urban forests present themselves as often fragmented and highly diverse in structure and composition, and so, potentially overlap with urban green spaces (UGS) or green-blue infrastructure (GBI) spatially, temporally, or ecologically (Ordóñez, 2021 and Table 1). With urban forests being connected to their wider physical (built) environment alongside social-institutional characteristics, e.g., property relationships, manifold nature-society interactions emerge (Ordóñez and Duinker, 2010).

**Table 1:** Competency questions to facilitate the identification of core concepts and relationships for guiding the further conceptualization of the proposed ontology. Answers to each question are based on expert knowledge and the analysis of the state-of-the-art. Core concepts to guide the further conceptualization of UF-NBS are printed in italics.

Competency question	Answer
What is urban forest?	*Urban forest* refers to tree-dominated ecosystems within cities (Berglihn and Gómez-Baggethun, 2021). This includes *forests*, *clusters or groups of trees*, as well as *solitary trees* in public and private spaces and across different types of urban green spaces such as *parks*, *gardens*, *on facades*, or *rooftops* (Endreny, 2018, FAO, 2016, Konijnendijk, 2003, Ordóñez, 2021).
What is urban green space (UGS)?	Here, *UGS* are referred to as natural or semi-natural land in urban areas, i.e., vegetated plots including agricultural areas, *lawns*, *forests*, *parks*, *gardens* (Collins et al., 2022, WHO Regional Office for Europe, 2016).
What is green-blue infrastructure (GBI)?	*GBI* is understood as natural or semi-natural *landscape element*s, that (may) form an interrelated green-blue network across different spatial scales (Langemeyer and Baró, 2021, Vlaamse Land Maatschappij, 2019).
What is a nature-based solution (NBS)?	*NBS* are *action*-oriented, cost-effective, locally adapted, systemic interventions supported by nature for a simultaneous provisioning of environmental, social, and economic *benefits* to build resilience (Almenar et al., 2021, Dumitru and Wendling, 2021, European Commission, 2015, Pauleit et al., 2017). NBS aim at the *protection*, *management*, or *restoration* of *ecosystem services* to provide benefits to society and biodiversity (Cohen-Shacham et al., 2016, Hanson et al., 2020). NBS may also be understood as an umbrella term that is related to and evolves neighbouring concepts, including urban forestry, UGS, and GBI, and *ecosystem services* (Dumitru and Wendling, 2021, Pauleit et al., 2017).
What is an urban forest as nature-based solution (UF-NBS)?	*UF-NBS* build on tree-based urban ecosystems to address societal challenges, and their importance for human well-being and biodiversity (Dumitru and Wendling, 2021). UF-NBS are closely related to the urban forest as a spatial entity, and to actions for the expansion, protection, and maintenance of the urban forest.
What are specific types of UF-NBS to be covered by the proposed UF-NBS typology?	The proposed typology needs to account for UF-NBS as spatial entities, and urban forest-related actions. UF-NBS to be covered include *urban forest*s, *tree clusters*, and types such as *street trees*, or *promenades,* as well as trees located in urban parks or gardens. UF-NBS as actions include *afforestation*, *reforestation*, tree *planting*, or the *monitoring* of trees and forests. In addition to these UF-NBS in a narrower sense, we also consider the spatial context of these UF-NBS as relevant. This particularly includes UGS, as potentially tree-dominated urban ecosystems (Ortí et al., 2022).
Which traits are relevant to describe UF-NBS?	Traits must be able to describe *spatial, morphological*, *structural*, *functional*, or *institutional* characteristics. Factors governing the biophysical properties of UF-NBS, i.e., *tree traits*, need to be considered. Covered traits must also be suitable to contextualize mediating factors. This includes spatial relationships, e.g., *adjacency.* Grouping principles applied in top-down typologies help to identify further traits.
What is a forest?	A *forest* may be understood as an *area* that is at least 0.5 ha in *size* (FAO, 2018), and that is covered by and thus dominated by trees.Janssen and Rosu (2015) suggest a *canopy cover* of at least 50 % for the delineation of such *treed areas*. This threshold may currently be observed, or yet to be reached.

At the same time, urban forests as nature-based solutions (UF-NBS) emphasize the importance of tree-based urban ecosystems for human health, human well-being, and biodiversity (Dumitru and Wendling, 2021). In line with nature-based solutions (NBS) as cost-effective, locally adapted, and systemic interventions for the provisioning of environmental, social, and economic benefits (Almenar et al., 2021, Dumitru and Wendling, 2021, European Commission, 2015, Pauleit et al., 2017), UF-NBS aim at the protection of existing tree stock, the optimization of ecosystem service delivery (Walters and Sinnett, 2021), the greening of cities, e.g., through tree planting initiatives (Doroski et al., 2020, Eisenman et al., 2021), and the engagement of communities to increase urban forest stewardship (Ordóñez, 2021).

Consequently, a typification of UF-NBS must not only embrace such kinds of interventions, but also needs to reflect on site-specific conditions and differences that result from the diverse structure and high spatial heterogeneity of urban forest (Münzinger et al., 2022).

The varied (semi-)natural elements forming UF-NBS deliver manifold benefits through providing ecosystem services, e.g., climate or air quality regulation, the provisioning of raw materials, food and water, or recreational and restorative potential (Ayala-Azcárraga et al., 2019; Berglihn and Gómez-Baggethun, 2021; Marando et al., 2019; Vieira et al., 2018). In so doing, UF-NBS help addressing 15 out of 17 sustainable development goals (Turner-Skoff and Cavender, 2019). This renders UF-NBS important tools to make cities more resilient, more liveable, and healthier (Endreny, 2018, FAO, 2016).

However, this ecosystem service delivery is mediated by urban morphology and form, including land-use heterogeneity, fragmentation, and use intensity, and subsequently, by the composition, shape, size, persistence, and interconnectivity of green elements (Alberti, 2005, Holt et al., 2015, Steenberg et al., 2015). Ecosystem service delivery of tree-based ecosystems is further mediated by the state and dimension of trees with respect to height, width, connectivity, and species diversity; soil conditions; the proximity to buildings and infrastructures; the location within street canyons or open structures of different size; accessibility; and tree maintenance (Cimburova and Berghauser Pont, 2021).

For a typification of UF-NBS, this calls once more for a close consideration of site-specific conditions and spatial and contextual relationships of UF-NBS with their physical, social, and ecological environment, to support the estimation of their material and immaterial benefits, and to make UF-NBS more comprehensive entities. It is furthermore argued that for best outcomes of UF-NBS action, a multifunctional perspective on UF-NBS should be adopted (Ordóñez, 2021). To do so, again, a systematic understanding of their (small-scale) impacts is needed.

### 1.1 Objectives of this paper

Against this background, this paper challenges the complexity of typifying UF-NBS that emerges from the spatial and structural diversity of urban forests, and from the manifold actions related to them, through a trait-based modelling approach. In trait-based modelling, traits are understood as morphological, physiological, phenological, biochemical, or structural characteristics of individual organisms with impact to their fitness or to ecosystem properties (Violle et al., 2007, Wellmann et al., 2018, Zakharova et al., 2019). Furthermore, Andersson et al. (2021) propose the concept of social-ecological traits, that “[…] shape […] ecological systems and linked human affordances […], and social systems by shaping the context of human activities and experiences”. Expanding on these notions, here, a trait is understood as a discernible aggregate feature of UF-NBS including spatial, morphological, contextual, structural, functional, and institutional characteristics.

Therefore, to advance the state-of-the-art, contingent on such traits, and complemented by semantic modelling, i.e., the systematic structuring of knowledge through the identification of relevant concepts and the modelling of their taxonomy, interrelationships, and properties (Villa et al., 2009), this paper develops a systematic, trait-based UF-NBS typology.

Thereby, in seeking to provide a common, more comprehensive understanding of UF-NBS, first, a conceptual UF-NBS model including a vocabulary of terms is being devised, to enable a trait-based description and to drive a subsequent definition of UF-NBS. The following section explores the state-of-the-art to support the development of this conceptual model. Second, as explained in the sections thereafter, through the operationalization of this conceptual model in form of an ontology, a machine-readable, machine-interpretable, re-usable and extendible knowledge representation is created (Gašević et al., 2006). In so doing, on the one hand, the conceptual UF-NBS model shall be transformed into a knowledge body that may be queried, and reasoned upon, using different software tools. On the other hand, formal anchor points for a later integration of knowledge and data shall be established, to facilitate UF-NBS assessment and governance, and to allow for the accommodation of a growing body of research.

## 2 Exploring state-of-the-art typologies for kick-starting ontology development

The implementation of the proposed UF-NBS typology requires an identification of relevant UF-NBS. As stated previously, UF-NBS are closely related with UGS, GBI, and NBS (cf. Table 1 for definitions). Hence, exploring typologies related to those concepts may assist in the elicitation of different UF-NBS types and traits, and in determining the role, part-taking, or overlaps of UGS, GBI, and NBS as contextual entities with UF-NBS. There are several typologies seeking to provide a systematic grouping of UGS, GBI and NBS, that recognize the multiple (co-)benefits provided by urban greenery and associated challenges. Broadly, these typologies may be grouped into *top-down* and *bottom-up* typologies.

*Top-down typologies* are commonly based on classification principles that reflect on spatial, physical, functional or governance characteristics for grouping UGS, GBI or NBS. The GREEN SURGE typology proposes various UGS types, e.g., “private, commercial, industrial, institutional UGS and UGS connected to grey infrastructure” or “parks and recreation”, and collates empirical evidence for the delivery of provisioning, regulating, cultural and supporting ecosystem services (Cvejić et al., 2015). Its focus is on spatial entities of UGS and associated benefits, thus neglecting NBS in the form of interventions. Such interventions are explored by Eggermont et al. (2015), suggesting a classification of NBS along three main categories: (i) minimal-intervention NBS seeking to maintain or improve the delivery of ecosystem services by given ecosystems; (ii) the development of management approaches for sustainable and multifunctional ecosystems; and (iii) the intrusive management or creation of novel ecosystems, inclusive of specific UGS types. Furthering these NBS categories, the ThinkNature typology recommends explicit actions and strategies for different types of UGS or desired benefits, including management practices, planning guidelines, monitoring actions, or restoration interventions (Nikolaidis et al., 2019). In a similar vein, the Nature4Cities typology employs an analytical framework that recognizes a level of human intervention for NBS; urban challenges; spatio-temporal scales; and physical environment (Nature4Cities, 2018). Embedded in this framework, Nature4Cities (2018) examines NBS actions and strategies as well as NBS construction and design principles.

In contrast, *bottom-up typologies* use trait-based modelling approaches to derive green elements as a function of local conditions and traits. For example, based on an ecosystem classification approach, Steenberg et al. (2015) propose a categorization schema of traits combined with hierarchical clustering for the identification of urban forest types in the city of Toronto. In their typology, traits include biophysical properties, e.g., grass or tree canopy cover, built environment factors such as building coverage, and social indicators. Similarly, Kok et al. (2016) elaborate a green infrastructure typology that abstracts land-cover, structural-morphological features, and spatial-configurational arrangements, such as solitary or clustered vegetational elements. Green infrastructure types, e.g., vegetated surfaces or intensive green roofs, are subsequently derived from the permutation of these traits.

When comparing these types of typologies broadly, it becomes clear that bottom-up examples tend to be based on specific traits, but lack an overarching action context or institutional aspects that are recognized as important governance challenges for NBS (Ordóñez, 2021). Contrary to that, top-down typologies tend to consider such a governance dimension, as well as manifold grouping principles, but lack to conceptualize mediating local conditions more closely. Hence, the integration of trait-based, bottom-up approaches with the grouping principles and the governance focus of top-down typologies is where the state-of-the-art is picked up in conceptualizing the proposed UF-NBS typology.

## 3 Methodology

**Fig. 1:**
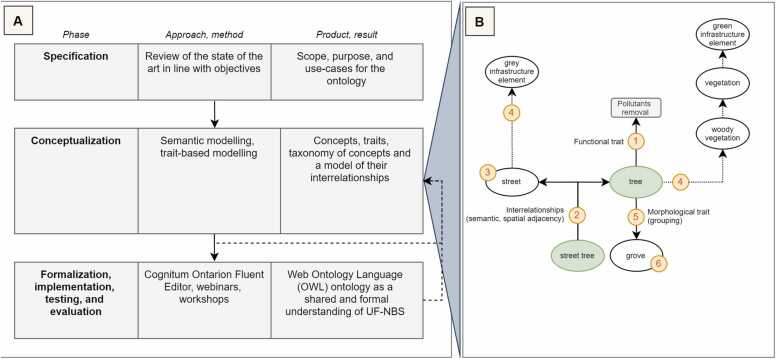
Building the proposed UF-NBS ontology comprises the phases of specification; conceptualization; and knowledge formalization, implementation, testing, and evaluation (A). The scope- and purpose-driven conceptualization of UF-NBS knowledge is a core activity of ontology building. Here, conceptualization is supported by a middle-out strategy as shown on the right (B). Ellipses in green exemplify an initial set of core concepts, with their characteristics and interrelationships being modelled, e.g., functional traits (1) or spatial relationships (2). Simultaneously, e.g., from expert knowledge, guided by competency questions, or through abstraction and generalization, further UF-NBS-relevant concepts are subsequently identified (3), resulting in a concept taxonomy (4). Similarly, through concept specification and detailing (5), further relevant concepts may also be derived (6). This process is iterative and is repeated until the conceptual model is in line with the scope and purpose of the ontology.

Semantic modelling for ontology implementation is also referred to as ontology building. There are several ontology building methodologies that typically comprise the specification of scope and purpose, including a review of existing knowledge; the conceptualization; and the formalization, implementation, testing, evaluation, and maintenance of knowledge (Gómez-Pérez et al., 2004; Uschold and King, 1995). The methodology for implementing the proposed UF-NBS typology as an ontology closely mirrors these steps (Fig. 1 A).

### 3.1 Specification

In line with the objectives of this paper, the proposed ontology’s scope and purpose is the implementation of a conceptual model on UF-NBS for promoting human health and well-being, including a reflection on the relationships between UF-NBS and NBS, as well as UGS and GBI as important enablers of citizen’s health and well-being. The review of existing knowledge was assisted by competency questions that recapitulate key questions for the discovery and structuring of knowledge (Sure et al., 2009). These competency questions were answered through exploring the state-of-the-art, including the typologies outlined above, as well as a review of 422 records on the benefits of UF-NBS for liveability, public health, and biodiversity (Scheuer et al., 2021a and Fig. S1). The development was further supported by expert knowledge on (urban) forestry, e.g., involving the European Forest Institute and the Croatian Forest Research Institute.

### 3.2 Conceptualization

The conceptualization phase corresponds to the actual semantic modelling process. Here, a middle-out strategy is followed (Fig. 1B). Supported by the exploration of the state-of-the-art as part of the specification phase, the middle-out strategy suggests the elicitation of an initial set of core concepts as a starting point (Gómez-Pérez et al., 2004, Uschold and Grüninger, 1996), with their taxonomy, interrelationships, and traits to subsequently be modelled. Simultaneously, through an abstraction and generalization of concepts, or through their further specification and detailing, additional relevant concepts may be identified, and added to the conceptual model as deemed necessary by the modeller (Fig. 1B). This process is repeated until the conceptual model meets the specified scope and purpose. The resulting conceptual UF-NBS model is subsequently formalized and implemented.

### 3.3 Formalization, implementation, testing, and evaluation

Knowledge formalization and implementation describes the process of transforming the conceptual UF-NBS model into formal knowledge by means of an ontology language. Here, the *Web Ontology Language (OWL) version 2* is chosen, as it provides a rich vocabulary for the modelling of concepts, properties and (taxonomic) relationships (Hitzler et al., 2012). The implementation is conducted using *Ontarion Fluent Editor* by *Cognitum*. This tool simplifies ontology building through the *Ontarion Controlled Natural Language* (OCNL), i.e., a comparatively easy to understand but machine-executable syntax that is internally translated into OWL constructs (Kapłański, 2011, Seganti et al., 2016).

Using *Ontarion Fluent Editor*, the so-implemented typology is continuously checked for syntactic correctness, i.e., that it remains computable for inference and reasoning, and based on test cases, evaluated for consistency, to ensure that it is in accordance with the conceptual UF-NBS model. A further evaluation of the implemented typology is conducted through a series of webinars and stakeholder workshops.

## 4 Results

Consultation of the state-of-the-art allowed answering a set of competency questions (Table 1) for kickstarting ontology building and knowledge conceptualization, i.e., to guide the elicitation and definition of core concepts, to aid in formulating their interrelationships, and thus, to structure UF-NBS knowledge.

**Fig. 2:**
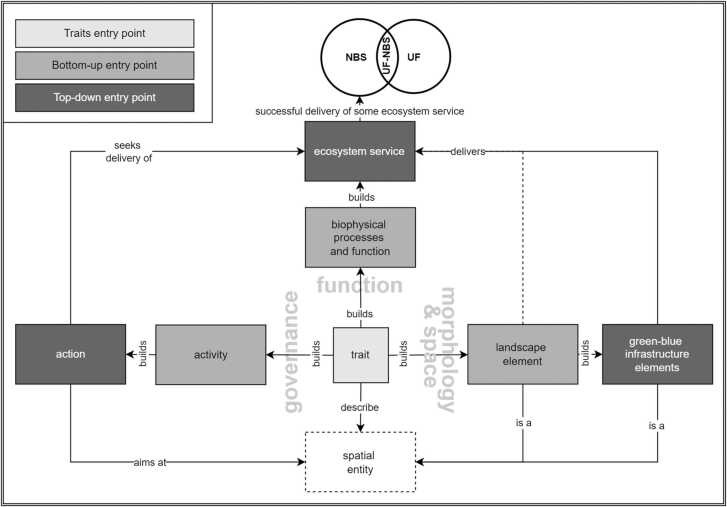
UF-NBS are conceptualized as the intersection of NBS with urban forest(try), and are considered multidimensional entities that embrace a governance, spatial-morphological, and functional dimension. Each dimension is specified based on traits. Within the governance dimension, traits build activities, that are, in turn, parts of actions. The spatial-morphological dimension comprises trait-based landscape elements, that serve as building blocks of GBI. GBI, in turn, correspond to spatial entities that may subsequently be detailed by further traits. The functional dimension devises a (potential) delivery of ecosystem services that is grounded in biophysical processes and functions, with ecosystem services delivery being considered key for successful NBS.

Overall, here, UF-NBS are defined as the formal intersection of NBS and the urban forest concept (Fig. 2). Therefore, their conceptualization is guided by an understanding of NBS as a broad umbrella term, aligned to urban forest(try). For this purpose, NBS are considered multidimensional entities, including the dimensions of (i) governance, i.e., a broad action and challenge orientation inclusive of institutional aspects, stewardship, participation etc.; (ii) function, i.e., a relation to biophysical processes, and ecosystem services delivery; and (iii) morphology and space, i.e., the adoption of spatial, contextual, and morphological characteristics (Fig. 2).

Subsequently, relationships are formalized to link the different dimensions. Actions are reckoned to target given spatial entities in seeking the delivery of benefits, thus associating governance action with the functional and the spatial-morphological dimension. These latter two dimensions are linked by viewing (semi-)natural landscape elements, and hence GBI elements, to (potentially) deliver ecosystem services as a function of biophysical processes, as mediated by their morphology, composition, site-specific conditions, and local spatial contexts.

Moreover, each of the NBS dimensions represents a rather generic notion, thus requiring further specification. Therefore, each dimension is understood as a “combination of properties”, or “composition of things”, respectively, that is constructed bottom-up through combining relevant concepts and traits. For example, afforestation action is understood to comprise tree planting activity. Likewise, trees are regarded as (green) landscape elements, i.e., as spatial entities that serve as building blocks of forest as a type of GBI.

There are three entry points emerging from this framing (Fig. 2): (i) a traits entry point as a “starting point” for the elicitation of traits to drive the trait-based modelling of UF-NBS inclusive of the postulated NBS dimensions; (ii) a “top-down” entry point that is related to ecosystem services as well as UF-NBS types identified a-priori, as modelling “end points”; and (iii) a “bottom-up” entry point, that is concerned with the description of the aforementioned “end points” with the help of traits. Therefore, this entry point puts the trait-based modelling into operation.

### 4.1 The traits entry point

The ontology proposes various traits for the expression of spatial context, morphology, form, and of physical, structural, functional, or institutional characteristics. A set of properties allows to express spatial context through spatial-topological relationships by stating adjacency, (full) enclosure, or part-taking of entities. Morphology and form may be conveyed in a two-fold manner: (i) through expressing the grouping of objects as solitary, linear, or clustered. For clustered arrangements, regular and irregular grouping patterns are considered to reflect on different vegetation structures (Lehmann et al., 2014); and (ii) through describing the composition of entities, e.g., being inclusive of trees, shrubs, water bodies, or amenity features. Traits for the description of physical location include being ground-based, being associated with support structures, e.g., potted, or being located on balconies and rooftops. Function is denoted through the contribution of entities to biophysical processes and/or functions, e.g., evaporation or active recreational use, and with ecosystem services delivery, e.g., regulation of air temperature and humidity or recreation, then being inferred from transitive relationships. Regarding governance and institutional context, e.g., institutional accessibility including public, semi-public, selective private, or temporally (un-)restricted access, and entitlement schemes—e.g., unrestricted public use, restricted collective use, or restricted private use—are formalized (Nissen, 2008). Further traits related to actions as a form of governance are devised to assert their spatial context, i.e., the target area of interventions, and to express involved activities.

### 4.2 The top-down entry-point

As stated above, the top-down entry point subsumes “end points” for modelling. Regarding ecosystem services, the ontology formalizes several supporting services, provisioning services, regulation and maintenance services, and cultural ecosystem services. Regarding actions and GBI elements as prospective UF-NBS, in line with the paper’s objectives, several types were identified:

• As actions: afforestation, reforestation, orchard restoration, enrichment planting, tree and forest monitoring, tree pruning, tree watering, and pest management.
• As spatial (GBI) entities: forests, riparian forests, nurseries, and forest plantations; elements of the urban food forest, e.g., orchards; elements associated with urban networks, e.g., street trees or tree alleys; greenery associated with buildings or built-up structures, e.g., green roofs, green facades, or atria; and UGS types such as parks, gardens, and cemeteries, that may pose specific or contextual UF-NBS entities, e.g., depending on their composition.

Furthermore, reflecting on the classification of NBS suggested by Eggermont et al. (2015), the typology proposes the grouping of actions depending on their outcome or intention, i.e., a grouping based on whether an action seeks to provide additional benefits through the construction of novel UF-NBS; the improvement of ecosystem service delivery of current UF-NBS through restoration; or the long-term safeguarding and maintenance of ecosystem service delivery through appropriate UF-NBS management (Smith et al., 2016).

### 4.3 The bottom-up entry point

In line with the notion of (UF-)NBS dimensions as “compositions of things”, the bottom-up entry point puts the trait-based modelling of concepts into operation. Thus, following the middle-out strategy, concepts are modelled bottom-up through the assignment of relevant combinations of spatial, morphological, functional, or institutional traits, and by their relationships with other concepts. Through a subsequent combination of suchlike constructed entities, increasingly higher-level concepts are devised, that may be re-used iteratively as building blocks for further entities, until the UF-NBS “end points” identified top-down are sufficiently defined.

With respect to the governance dimension, building blocks of actions are defined in the form of so-called activities. Exemplarily, activities include the planting of trees or shrubs, pest control, pruning, or monitoring. In a similar way, building blocks of the spatial-morphological dimension encompass various green, blue, and grey (built-up) landscape elements. Green landscape elements comprise vegetational elements, i.e., woody and non-woody vegetation, and thus grass, shrub, or trees. Rather than relying on this generic and abstract notion, traits such as genus or species, age, leaf area index, etc. may also be designated. Higher-level building blocks are then derived through a further allocation of traits. For instance, a single tree is derived by attributing a solitary grouping to the tree concept, whilst a grove is devised as a clustered group of trees. Edible plants, including fruit trees, may be inferred from food production as functional trait. Blue landscape elements conceptualize watercourses, e.g., rivers or canals, or permanent bodies of standing water, such as ponds. Grey landscape elements comprise elements from the built environment, including houses or (multistorey) buildings, urban network elements such as paths, tracks, or streets, and selected amenity features, e.g., sports fields, playgrounds etc., with functional or institutional traits being added as needed, e.g., to indicate accessibility or entitlements of use.

### 4.4 The synergy of the three perspectives

In the following, exemplified for (riparian) forests, single street trees, neighbourhood green spaces, tree watering, and afforestation, it will be described how all entry points are brought together, and therefore, how traits, and thus the trait-based vocabulary provided by the proposed ontology, assist in a systematic characterisation of UF-NBS.

**Fig. 3:**
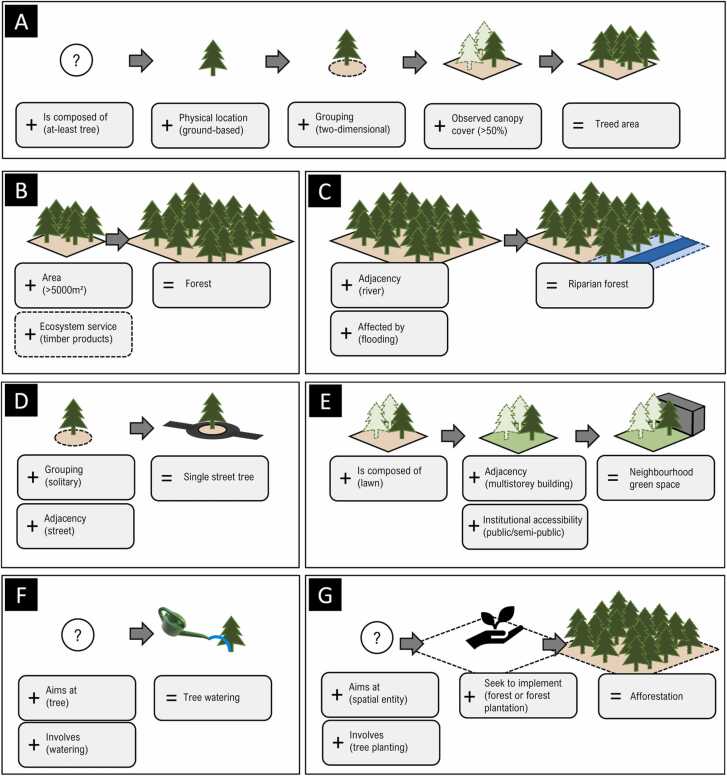
Definition of UF-NBS through trait-based modelling. Treed areas are derived through the assignment of traits describing composition, physical location, grouping of elements, and canopy cover; here, either observed values or potential canopy cover yet to be reached may be considered (A). Forests are in turn derived from treed areas by adding a size constraint, with functional traits being added if applicable (B). Different types of forest may subsequently be distinguished. Here, riparian forest is derived from added traits describing adjacency and impact by physical phenomena (C). Similarly, through the re-use of building blocks, single street trees may be inferred from ground-based trees, with traits added to express solitary grouping and adjacency to street (D). Through the description of spatial context, and by capturing institutional characteristics, further types of GBI, e.g., neighbourhood green spaces, may be conceptualized. Those may pose UF-NBS themselves or provide site-specific context to UF-NBS entities. (E). Actions are formalized based on involved activities, e.g., watering, and by pointing at the entities they are aimed e.g., tree, here, resulting in tree watering as UF-NBS action. (F). Similarly, by targeting a spatial entity (of any kind) for planting of trees, and by additionally stating the goal of this activity as seeking to implement a forest, afforestation may be denoted (G).

Following the competency questions (Table 1), a forest is understood as a treed area of specific size. Consequently, first, treed area is conceptualized as being composed of trees, that are ground-based, grouped in a two-dimensional manner, and arranged so that at least 50 % of the area is (or will potentially be) covered by tree canopy (Fig. 3 A). Subsequently, the forest definition is completed by re-using the treed area concept, and by adding a size constraint of at-least 0.5 ha. Further traits, e.g., functional traits such as the production of timber, may be added if applicable for the entity in question (Fig. 3B). Fig. 3 C exemplifies how further types of forest may in turn be formalized. For instance, here, riparian forest is derived from forest by adding traits describing adjacency to a river, and impact by flooding as a physical phenomenon. Re-using known concepts as building blocks, single street trees may be described as ground-based, solitary trees adjacent to streets (Fig. 3D). Similarly, neighbourhood green spaces may be conceptualized, e.g., as compositions of different types of green landscape elements such as lawns and trees, and with further spatial-topological (adjacency) and institutional (institutional accessibility) traits being added (Fig. 3E). Depending on their morphological characteristics, such entities may subsequently pose UF-NBS themselves, or may contextualize site-specific conditions as important mediators of UF-NBS function. Likewise, using activities as building blocks, actions as types of UF-NBS are expressed by denoting their goals and/or their spatial context. For instance, tree watering as UF-NBS action is formalized by linking watering activity to trees (Fig. 3 F), and afforestation action is devised to comprise tree planting activity that is aimed at a given spatial entity to implement a forest (Fig. 3 G).

**Fig. 4:**
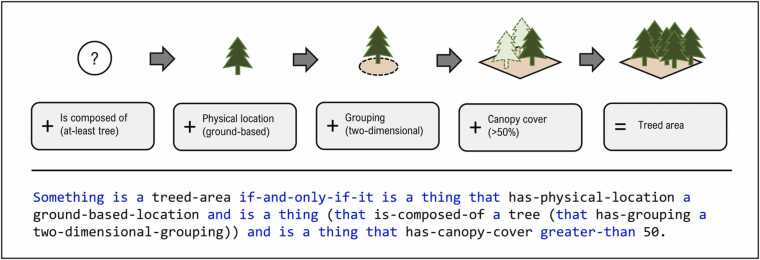
Formalization of conceptual UF-NBS knowledge using OCNL, exemplified for the treed area concept. OCNL syntactic elements are printed in blue, whereas devised traits and concepts, i.e., the vocabulary as provided by the proposed typology, is printed in black.

The so-conceptualized UF-NBS model was subsequently formalized and implemented using OCNL. As exemplified in Fig. 4, OCNL provides pre-defined syntactic elements, printed in blue, that are used in conjunction with the self-defined, trait-based UF-NBS vocabulary, printed in black, to formally express UF-NBS knowledge (Fig. 4). The complete implementation, including a full listing of OCNL statements, is described in more detail in Scheuer et al. (2021b).

**Fig. 5:**
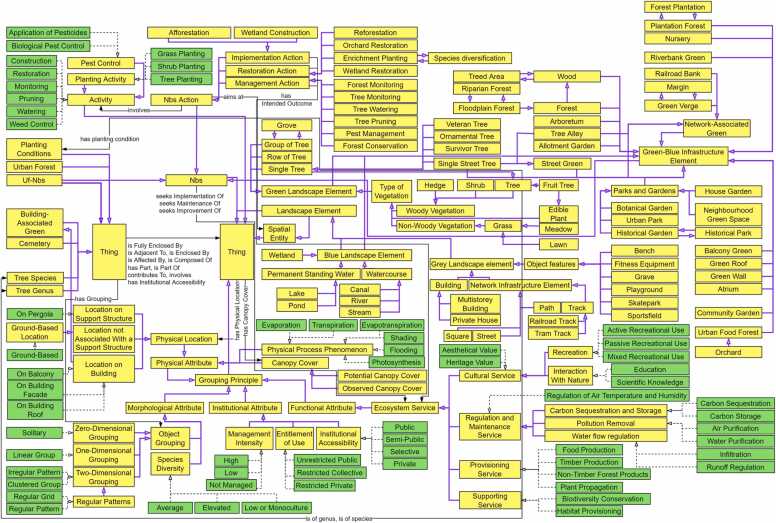
Semantic network of the proposed UF-NBS ontology. Concepts are visualized as yellow rectangles, and instances of concepts as green rectangles. Coloured edges indicate semantic interrelationships. Notably, purple edges denote taxonomic relations. Membership is indicated by dashed black edges, and formalized traits by solid black edges. Please refer to Fig. S2 for an enlarged version.

The implemented ontology is visualized in Fig. 5 in the form of a semantic network, i.e., a (directed) graph structure depicting concepts, their properties, and (taxonomic) interrelationships. Here, concepts are visualized as yellow rectangles. Green rectangles denote instances of concepts, e.g., types of ecosystem services or specific trait expressions such as ground-based, with dashed black edges indicating their corresponding concept membership. Purple edges show taxonomic and semantic relationships, e.g., equivalency, and solid black edges correspond to formalized traits.

To conclude ontology building, the implemented ontology was sought to be evaluated in a series of virtual events, with particular focus on the underlying conceptual UF-NBS model. First, in a set of webinars, initial feedback was collected from consortium members, to ensure a shared understanding of concepts. Second, to obtain wider critique on the devised conceptual model, a public workshop was held. For this event, a broad audience of 119 stakeholders registered with institutional backgrounds from city administration, education and research, environmental networks, to environmental charities. Stakeholders were invited to discuss the trait-based approach, as well as potential avenues for dissemination and re-use. Thereby, further potentially relevant traits and UF-NBS types could be identified (Fig. S3). Following the middle-out strategy, these candidate concepts may serve as core concepts, i.e., starting point, in a subsequent ontology building iteration.

## 5 Discussion and conclusions

By developing an ontology for the typification of UF-NBS, the work described in this paper is seen to advance the state-of-the-art in distinct ways. First, in contrast to rather narrative/textual definitions, trait-based and semantic modelling was used to approach the grouping of UF-NBS. By the simultaneous consideration of top-down and bottom-up approaches, a vocabulary of terms was elaborated that, based on conceptualized traits, enables the expression of defining spatial-morphological, functional, and institutional characteristics, thereby driving the systematization of UF-NBS knowledge.

In so doing, first, on the one hand, a consensual understanding of UF-NBS terms including their interrelationships is being developed, that may foster knowledge transfer and help bridging language or cultural barriers (Taylor and Hochuli, 2017). Similarly, (interdisciplinary) stakeholder discussion and decision-making may be facilitated by such a shared understanding. On the other hand, by allowing for an expression of site-specific conditions and spatial context, local factors mediating UF-NBS function and associated benefits may be reflected more systematically, with potential overlaps of UF-NBS with UGS and GBI being uncovered. By further expressing individual UF-NBS properties regarding vegetational structure, choice of tree species, tree age, or amount of greenery, etc., standardized UF-NBS inventories may be built that, based on a common language, depict the actual heterogeneity and diversity of UF-NBS and their embedding into the urban fabric to assist assessment, management, planning, and informed decision-making (Zürcher, 2017) as key to sustainable urban forestry (Dwyer et al., 2003). Thereby, the perception of UF-NBS as social-ecological systems (SES) is also being stressed. SES characterize UF-NBS not only by their natural components that govern biophysical functions and processes but emphasize the interactions and mutual impacts of these natural components with a co-evolving, multi-actor social subsystem (Fischer, 2018, Vogt, 2020). In this regard, as highlighted by Andersson et al. (2021), social-ecological traits mediate social experiences and human affordances, thus, the presence of features like playgrounds or sports facilities is seen to embody associated recreative benefits. In line with calls to establish broader definitions of GBI that focus more closely on the interrelationships between the natural and built environment in the production of ecological or societal benefits (Grabowski et al., 2022), the devised work adequality formalizes such transitive dependencies of form and function.

Second, through the implementation of this shared, conceptual understanding as an ontology, a formal, extendible, machine-readable, and machine-interpretable knowledge representation is created, that permits conducting reasoning and inference on UF-NBS knowledge such as the aforementioned inventories. In so doing, e.g., fuzzy relationships that result from underlying traits may be identified. For instance, depending on morphology, form, and institutional characteristics, spatial entities may pose polymorphic subjects that correspond to several UF-NBS types simultaneously, thus speaking to their multifunctional character (Ordóñez, 2021). By this means, rather generic land-use categories such as “urban green space” may be augmented by inferred (polymorphic) UF-NBS types and their respective traits, to support modelling of SES and the assessment of site-specific impacts. Thereby, also opportunities for learning, knowledge exchange, and sustainable management are opened. For example, factors for success or failure of UF-NBS may be more readily identified based on traits, thus assisting in the optimization of UF-NBS selection and design to obtain more-sustainable solutions to socio-environmental challenges.

In perspective, starting from there, knowledge-based decision-support tools may then be devised. However, to support such broader scopes, a refinement particularly of governance concepts will be required, e.g., to cover aspects such as participation, cost(-effectiveness), or actions aiming at the change of certain traits, e.g., accessibility. The ontology’s scope may also be furthered, and hence, its re-usability increased, by an extension with a technological dimension, that emphasizes how technology mediates social-environmental interactions and alters human agency (Ahlborg et al., 2019). In so doing, the ontology may be aligned with other trait-based frameworks or third-party ontologies, thereby, promoting the discovery of relevant knowledge and data. For example, Lausch et al. (2016) review trait-based indicators of forest health, stress and disturbances, and Vogt (2020) summarizes traits on the sustainability of urban forests related to vegetational characteristics and community and resource management. Battle and Kolas (2012) propose the GeoSPARQL ontology for the semantic representation and querying of geospatial data, and Aldana-Martín et al. (2022) propose the RESEO earth observation ontology to formalize remote-sensing data and products. In this regard, through included tree traits, the ontology already provides anchor points for the integration of databases on tree-related impacts such as i-Tree (Nowak et al., 2018). Thereby, linkage of relevant data, and thus, impact assessment shall be facilitated.

However, there are limitations to the presented approach. Particularly when compared to top-down typologies, a lack of distinguishing traits or shared understanding may result in a limited expressiveness. For example, due to these reasons, pocket parks as frequently mentioned elements of top-down typologies could not be considered. On a more general note, this touches on issues of scalability, that needs to be critically reflected considering the desired scope. As mentioned, the typology, and thus, the provided vocabulary of terms, focus primarily on (compositions of) UF-NBS entities for the delivery of human health and well-being benefits. Therefore, the ontology is directed at individual or grouped entities, e.g., trees or tree lines, and at compositions thereof, e.g., forests or urban parks, that correspond to scope-dependent, top-down-derived modelling “end-points”. Although the grouping principles outlined in the typology may also be applied to these entities, thereby deriving compositions at increasingly smaller spatial scales, e.g., city districts, the vocabulary of terms is seen to lack the required expressiveness to do so systematically, e.g., regarding use of built-up land etc.

Further limitations arise from certain open-ended specifications of UF-NBS traits. This applies, e.g., to the canopy cover threshold used in the definition of treed areas, and subsequently, forest. The chosen value is significantly higher when compared to the definition by FAO (2018). However, it approximates closed forest (>40 % canopy cover; Colson et al., 2009) and the UNESCO forest terminology (>60 % canopy cover; cf. Romijn et al., 2013), and with respect to the urban context that is in focus, the threshold chosen appears to be rather reasonable, with an average canopy of already 56 % reported for (European) UGS (Ortí et al., 2022). Clearly, these thresholds highlight the fuzziness of concepts. For example, changes in forest stock that result from differing thresholds, or therefore, forest definitions, could be explored by their adaptation. Moreover, in the case of UGS, e.g., it is only expressed that these concepts feature a certain institutional accessibility, but no specific mode of accessibility is universally defined. This is because it may only be possible to express such traits for actual UF-NBS instances. To exemplify, looking at examples of UGS in Berlin, Germany, there are those featuring public, free, and temporally unrestricted access (e.g., Treptower Park), whereas others feature public, free but temporally restricted access (e.g., Tempelhofer Feld), and yet others featuring public but temporally restricted and at-cost access (e.g., Gärten der Welt). This emphasizes once more that for the production of benefits, such site-specific, local conditions must be considered, as they may vary significantly between different instances of UF-NBS. It has been shown that the proposed approach allows for their consideration, and in so doing, provides anchor points for more specific research.

## Declaration of Competing Interest

The authors declare that they have no known competing financial interests or personal relationships that could have appeared to influence the work reported in this paper.
