# Supplementary material for: A trait-based typification of urban forests as nature-based solutions
Source: Urban For Urban Green. 2022 Dec;78:None. doi: 10.1016/j.ufug.2022.127780 (PMC9746330; doi:10.1016/j.ufug.2022.127780)
Supplement: Supplementary file 1 — Supplementary material. [file mmc1.docx]

TITLE-ABS-KEY(urban) AND TITLE-ABS-KEY(tree OR forest OR nature) AND TITLE-ABS-KEY('?'€'?'based-solution'?'€'?')AND TITLE-ABS-KEY(biodiv* OR health OR well-being OR livability)

TITLE-ABS-KEY(urban) AND TITLE-ABS-KEY(tree OR forest)AND TITLE-ABS-KEY(planning OR '?'€'?'green-infrastructure'?'€'?') AND TITLE-ABS-KEY(biodiv* OR health OR well-being OR livability)

TITLE-ABS-KEY(urban) AND TITLE-ABS-KEY(tree OR forest)AND TITLE-ABS-KEY(ecosystem W/5 service) AND TITLE-ABS-KEY(biodiv* OR health OR well-being OR livability)

**Figure S1.** *Search strings used for the identification of records on UF-NBS for promoting urban liveability, human health, and well-being. Initially, based on these search strings, 1290 records were queried from the SCOPUS database in August 2019. Following a first screening for availability and eligibility, 771 records could be accessed, from which a total of 422 records were reviewed in-depth following the evaluation of further eligibility criteria.*
