# Supplementary material for: A trait-based typification of urban forests as nature-based solutions
Source: Urban For Urban Green. 2022 Dec;78:None. doi: 10.1016/j.ufug.2022.127780 (PMC9746330; doi:10.1016/j.ufug.2022.127780)
Supplement: Supplementary file 2 — Supplementary material. [file mmc2.docx]

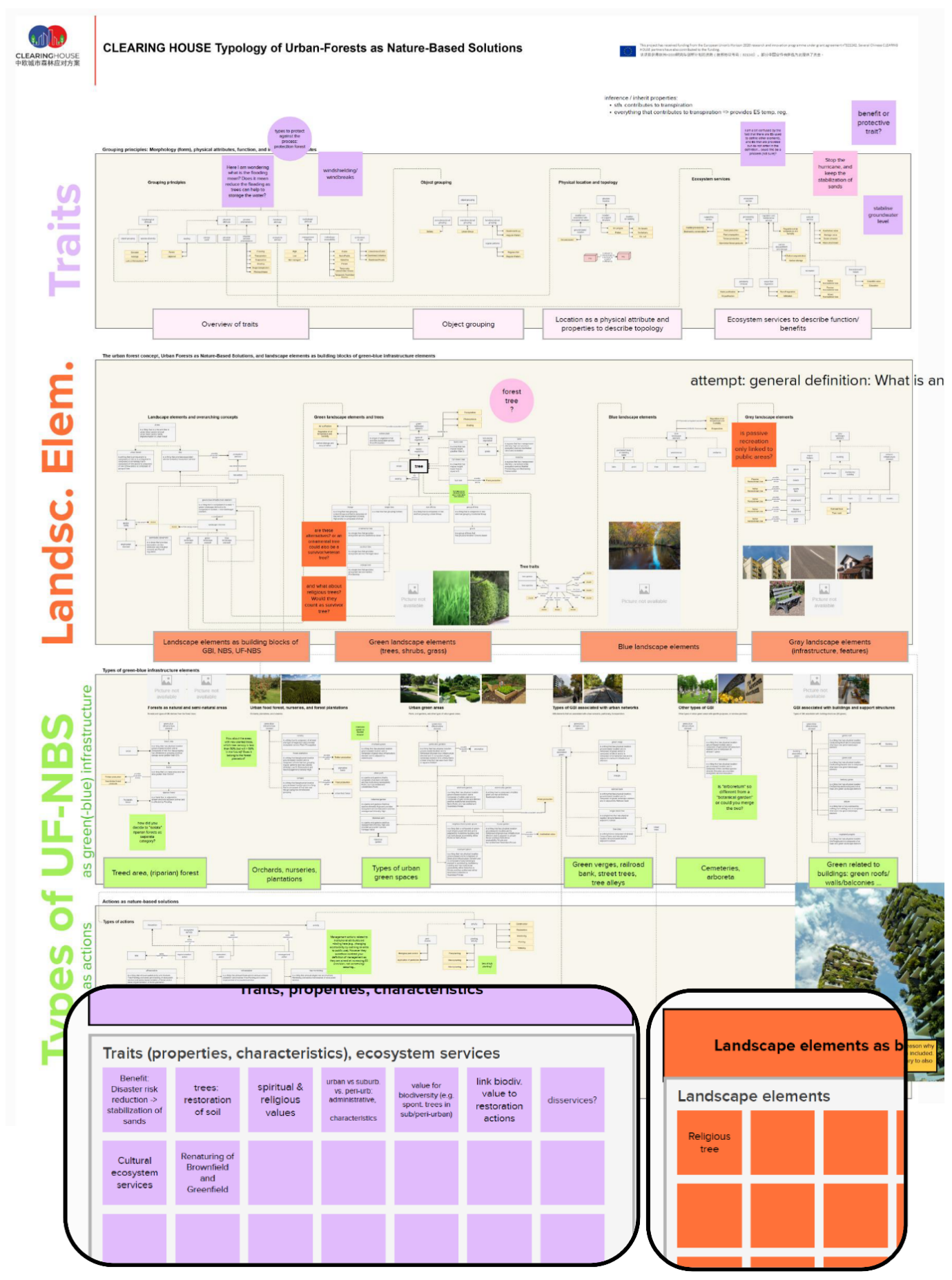


**Figure S3.** *Evaluation of the proposed UF-NBS typology with the help of a collaborative online whiteboard, as part of an online workshop. In this virtual event, a particular focus was on the discussion of the underlying conceptual UF-NBS model, including devised traits and types of UF-NBS types derived through trait-based modelling, and semantic modelling, respectively. On the whiteboard, the feedback of stakeholders was collected for future reference. Against the Sino-European background of the project this work results from, several comments emphasized the importance of religious and spiritual values, and suggested a closer consideration of corresponding landscape elements, e.g., religious trees. Similarly, culturally important concepts could be elicited for future integration, e.g., (often man-made) Fengshui woodlands. In this regard, the particular values attached to such entities need to be conceptualized further. Further points for revision include a closer conceptualization of UF-NBS for disaster risk reduction, or the (biodiversity) benefits delivered through spontaneous vegetation, e.g., on brownfields.*
