# Supplementary figures and images for: A trait-based typification of urban forests as nature-based solutions
Source: Urban For Urban Green. 2022 Dec;78:None. doi: 10.1016/j.ufug.2022.127780 (PMC9746330; doi:10.1016/j.ufug.2022.127780)

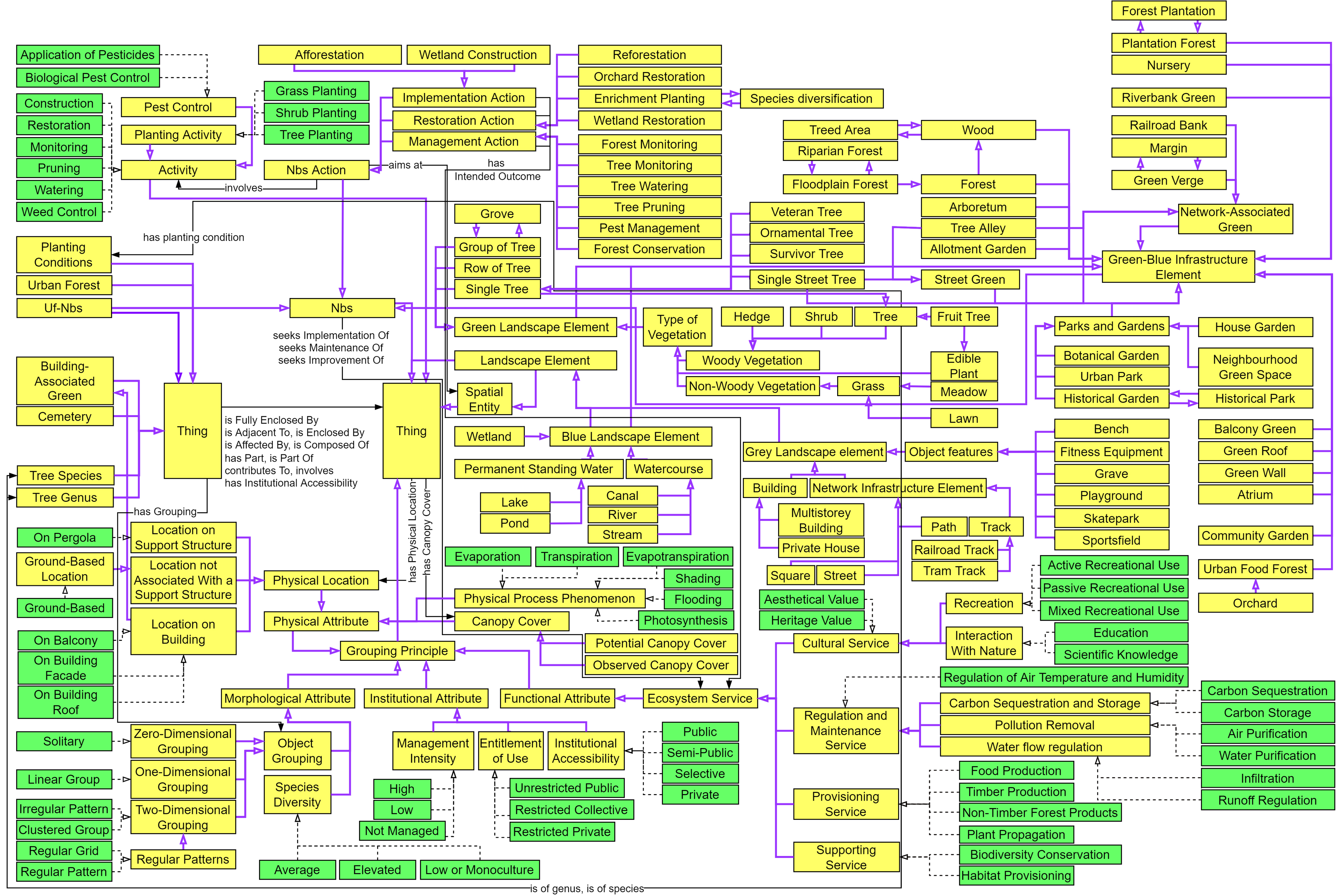

Supplement: Supplementary file 3 — Supplementary material. [file mmc3.jpg]
